# Supplementary material for: Perinatal Semaglutide Treatment Improves Maternal Health and Mitigates Offspring Metabolic Dysfunction in a Mouse Model of Maternal Obesity
Source: bioRxiv. 2026 Jun 11:2026.06.07.730723. Preprint. [Version 1] doi: 10.64898/2026.06.07.730723 (PMC13278011; doi:10.64898/2026.06.07.730723)
Supplement: Supplementary file 1 [file media-1.pdf]

Table S1. Mouse qPCR primer sequences for hepatic gene expression analysis.

Gene	Primer	Sequence (5'→3')
36b4	Forward	GGTGCCTCTGGAGATTTTCG
36b4	Reverse	CACTGGTCTAGGACCCGAGAAG
Srebp1c	Forward	GGAGCCATGGATTGCACATT
Srebp1c	Reverse	GGCCCGGGAAGTCACTGT
Acaca	Forward	GACAGACTGATCGCAGAGAAAG
Acaca	Reverse	TGGAGAGCCCCACACACA
Fasn	Forward	GCTGCGGAAACTTCAGGAAAT
Fasn	Reverse	AGAGACGTGTCACTCCTGGACTT
Scd1	Forward	CCGGAGACCCCTTAGATCGA
Scd1	Reverse	TAGCCTGTAAAAGATTTCTGCAAACC
Atgl	Forward	GCCTCCTTGGACACCTCAATAA
Atgl	Reverse	CTTCCTCGGGGTCTACCACA
Ppara	Forward	CACCTGCAGAGCAACCATC
Ppara	Reverse	CCGAAGGTCCACCATTTTT
Cpt1a	Forward	TGAGTGGCGTCCTCTTTGG
Cpt1a	Reverse	CAGCGAGTAGCGCATAGTCATG
Mcad	Forward	TTACCGAAGAGTTGGCGTATG
Mcad	Reverse	ATCTTCTGGCCGTTGATAACA
Acox1	Forward	CAGCAGGAGAAATGGATGCA
Acox1	Reverse	GGGCGTAGGTGCCAATTATCT
Cd36	Forward	TGTGTTTGGAGGCATTCTCA
Cd36	Reverse	TTTTGCACGTCAAAGATCCA
Cidea	Forward	CTCCGAGTACTGGGCGATAC
Cidea	Reverse	ACCAGCCTTTGGTGCTAGG
Fsp27	Forward	GGCTCACAGCTTGGAGGA
Fsp27	Reverse	CTCCACGATTGTGCCATCT
Plin2	Forward	GCTGCGGAAACTTCAGGAAAT
Plin2	Reverse	AGAGACGTGTCACTCCTGGACTT
Dgat2	Forward	AGCTGGTGAAGACACACAACC
Dgat2	Reverse	TGATGATAGCATTGCCACTCC
Sirt1	Forward	ATCGGCTACCGAGACAAC
Sirt1	Reverse	GTCACTAGAGCTGGCGTGT
Tnfa	Forward	AGCACAGAAAGCATGATCCG
Tnfa	Reverse	CCCGAAGTTCAGTAGACAGAAGAG
F4/80	Forward	CTTTGGCTATGGGCTTCCAGTC
F4/80	Reverse	GCAAGGAGGACAGAGTTTATCGTG
Il1b	Forward	GAATGACCTGTTCTTTGAAGTT
Il1b	Reverse	TTTTGTTGTTCATCTCGGAGCC
Timp1	Forward	CCTTTGCATCTCTGGCATCT
Timp1	Reverse	CTCGTTGATTTCTGGGGAAC

Supplementary Figure 1. Maternal food intake during treatment and pregnancy phases.

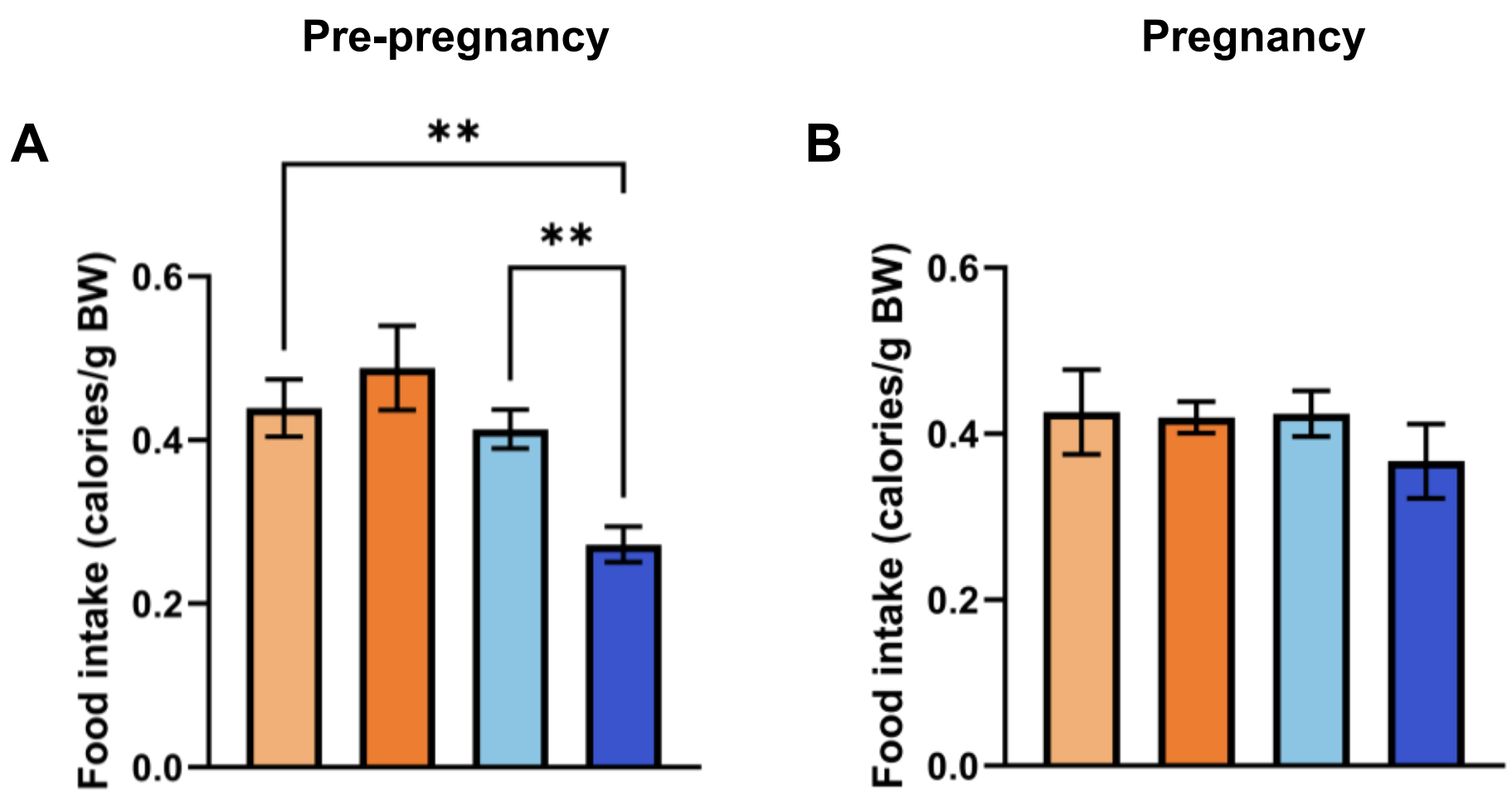

Supplementary Figure 1. Maternal food intake during treatment and pregnancy phases. (A) Food intake during the pre-pregnancy treatment phase (day 5). (B) Food intake during pregnancy at embryonic day (E)10.5. Data are presented as mean ± SEM (*n* = 10–12 chow, *n* = 15–16 HFD). **P ≤ 0.01.

Supplementary Figure 2. Pregnancy outcomes and placental morphology at E17.5.

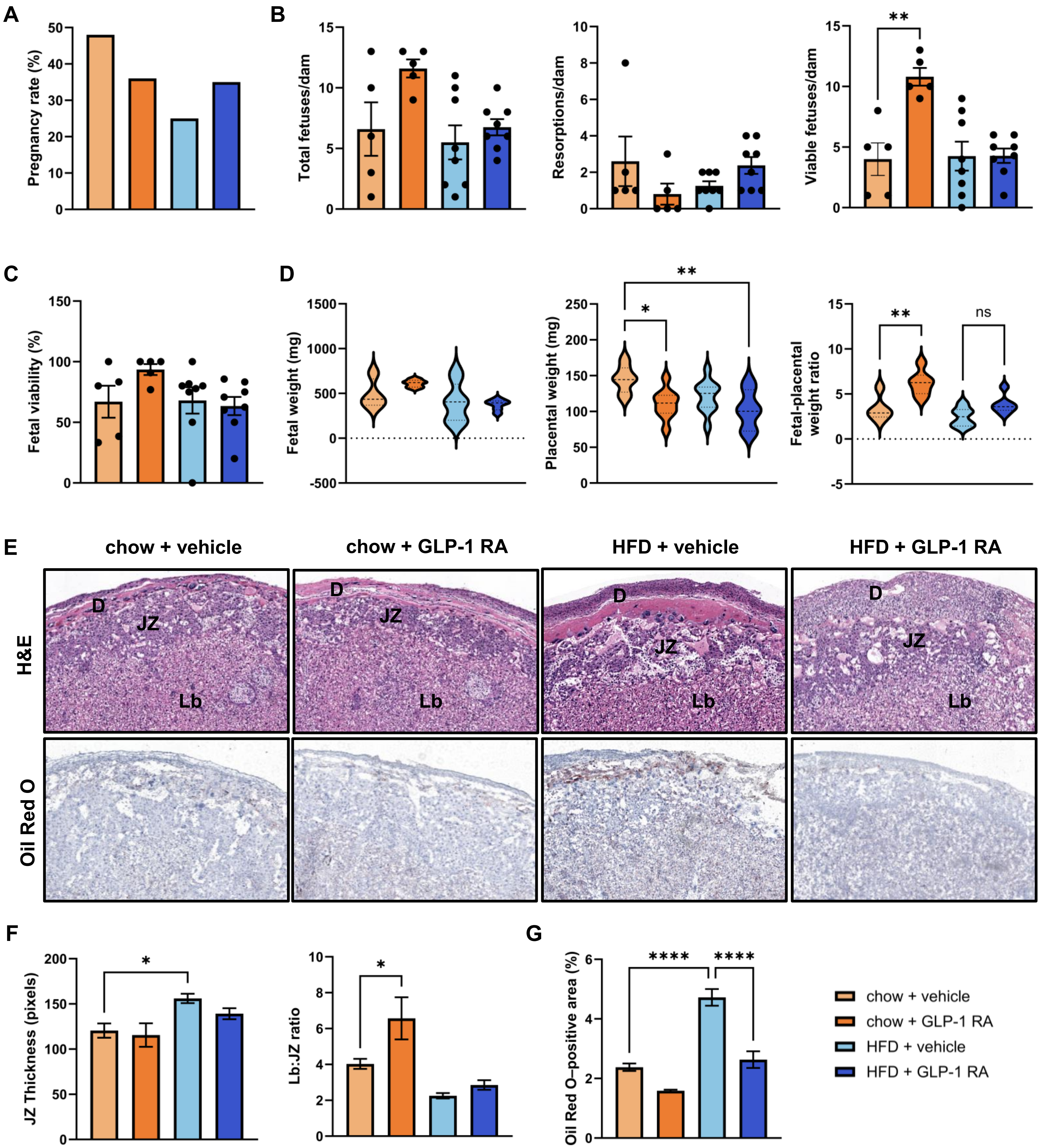

Supplementary Figure 2. Pregnancy outcomes and placental morphology at E17.5. (A) Pregnancy rate. (B) Total fetuses per dam, resorptions per dam, and viable fetuses per dam. (C) Fetal viability (%). (D) Fetal weight, placental weight, and fetal-to-placental weight ratio at embryonic day (E)17.5. (E) Representative hematoxylin and eosin (H&E, top) and Oil Red O (ORO, bottom) staining of placenta. (F) Junctional zone (JZ) thickness and labyrinth-to-junctional zone (Lb:JZ) ratio. (G) Placental ORO–positive area. Lb, labyrinth; JZ, junctional zone. Data are presented as mean ± SEM (chow: *n* = 5 litters; HFD: *n* = 8 litters). Pregnancy rate was analyzed using Fisher's exact test; all other comparisons were analyzed using one-way ANOVA. *P ≤ 0.05, **P ≤ 0.01, ****P ≤ 0.0001.

Supplementary Figure 3. Glucose and insulin tolerance in female F1 offspring.

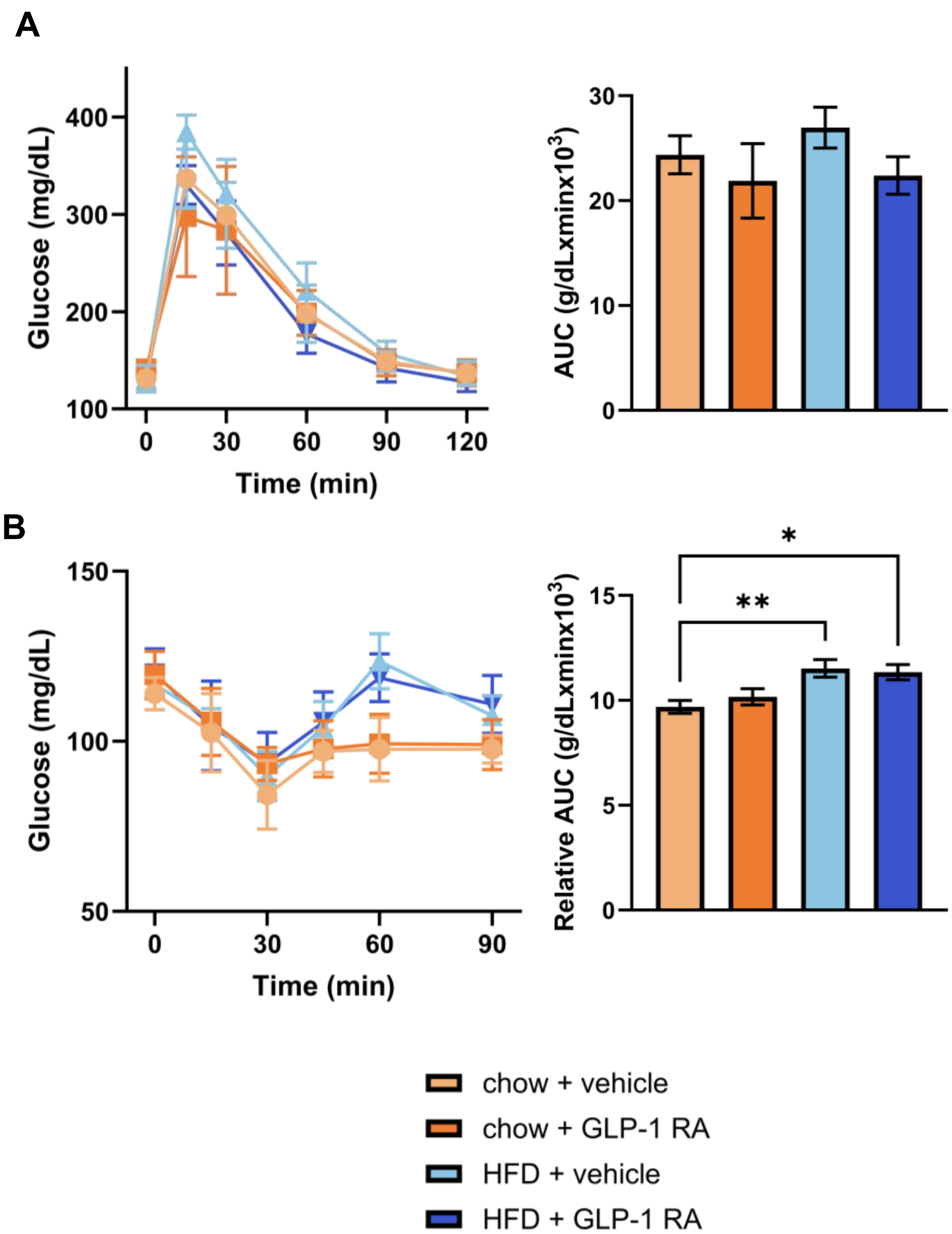

Supplementary Figure 3. Glucose and insulin tolerance in female F1 offspring. (A) Glucose tolerance test (GTT) at 8 weeks of age and insulin tolerance test (ITT) at 12 weeks of age in female F1 offspring. Area under the curve (AUC) for GTT and relative AUC for ITT are shown. Data are presented as mean ± SEM (*n* = 6–9 female offspring).
